# Burkholderia Lethal Factor 1, a Novel Anti-Cancer Toxin, Demonstrates Selective Cytotoxicity in MYCN-Amplified Neuroblastoma Cells

**DOI:** 10.3390/toxins10070261

**Published:** 2018-06-27

**Authors:** Aleksander Rust, Sajid Shah, Guillaume M. Hautbergue, Bazbek Davletov

**Affiliations:** 1 Structural and Molecular Biology, Division of Biosciences, Faculty of Life Sciences, University College London, London WC1E 6BT, UK; 2 Department of Biomedical Science, University of Sheffield, Firth Court, Western Bank, Sheffield S10 2TN, UK; sajidsw1@hotmail.com; 3 Sheffield Institute for Translational Neuroscience, Department of Neuroscience, University of Sheffield, 385a Glossop Road, Sheffield S10 2HQ, UK; g.hautbergue@sheffield.ac.uk

**Keywords:** ribosome-inactivating protein, BLF1, eIF4A, MYCN, cancer, neuroblastoma, apoptosis

## Abstract

Immunotoxins are being investigated as anti-cancer therapies and consist of a cytotoxic enzyme fused to a cancer targeting antibody. All currently used toxins function via the inhibition of protein synthesis, making them highly potent in both healthy and transformed cells. This non-specific cell killing mechanism causes dose-limiting side effects that can severely limit the potential of immunotoxin therapy. In this study, the recently characterised bacterial toxin Burkholderia lethal factor 1 (BLF1) is investigated as a possible alternative payload for targeted toxin therapy in the treatment of neuroblastoma. BLF1 inhibits translation initiation by inactivation of eukaryotic initiation translation factor 4A (eIF4A), a putative anti-cancer target that has been shown to regulate a number of oncogenic proteins at the translational level. We show that cellular delivery of BLF1 selectively induces apoptosis in neuroblastoma cells that display MYCN amplification but has little effect on non-transformed cells. Future immunotoxins based on this enzyme may therefore have higher specificity towards MYCN-amplified cancer cells than more conventional ribosome-inactivating proteins, leading to an increased therapeutic window and decreased side effects.

## 1. Introduction

A number of protein synthesis-inhibiting toxins isolated from plants (e.g., saporin) or bacteria (e.g., diphtheria toxin) are being investigated for use in cancer therapy as they are highly toxic to mammalian cells [1,2]. One of the major characteristics of these enzymatic toxins is that their catalytic nature conveys an extraordinarily high potency not possible with small molecules [3]. However, the non-selective toxic mechanism of protein synthesis inhibition means that these toxins are highly potent to all cell types. Re-targeting to specific cancer cells is, therefore, necessary and requires fusion of the toxin to either an antibody (or antibody fragment), or ligand specific for receptors highly expressed on the cancer cell surface [4]. Despite such targeting, low level presence of the receptors on healthy cells and non-specific cellular uptake are known to cause side effects such as vascular leak syndrome and hepatotoxicity for every targeted toxin developed to date. Toxins that maintain high potency but have a toxic mechanism more selective towards transformed cells would, therefore, be of high interest in targeted toxin design.

Burkholderia Lethal Factor 1 (BLF1) is a monomeric toxin from the bacterium *Burkholderia pseudomallei* that was characterised in 2011 and has been proposed as a possible anti-cancer agent [5,6]. As with saporin and diphtheria toxin, BLF1 has been shown to cause cytotoxicity with high potency by irreversible inhibition of translation initiation and subsequent protein synthesis. BLF1 targets the translation initiation phase of translation via inactivation of the eukaryotic initiation translation factor 4A (eIF4A) through deamidation of the glutamine 339 [4]. Translation initiation is the rate limiting step of protein synthesis and is up-regulated in most cancers, contributing to increased levels proteins involved in a number of oncogenic processes [7]. During translation initiation, the eIF4F complex is assembled from the mRNA cap-binding protein eIF4E, the RNA-helicase eIF4A and the scaffold protein eIF4G. These compose part of the 43S pre-initiation complex involved in scanning the 5′ UTR for the translation start [8]. The eIF4F complex acts as a central node upon which a number of oncogenic signalling pathways (e.g., Ras, PI3K/AKT/mTOR and Myc) converge [9]. eIF4A is an RNA-helicase that resolves the secondary structures found in the 5′ UTR of mRNAs. This is necessary for scanning of the 5′ UTR by the pre-initiation complex to reach the translation start site [8]. It has recently been shown that a subset of mRNAs with long and complex 5′UTRs that contain G-quadruplex secondary structures have high dependence on eIF4A activity [10]. A number of important proto-oncogenes such as c-Myc, cell cycle regulators and survival proteins have been shown to be regulated by this mechanism and inhibition of eIF4A leads to the preferential down-regulation of these proteins, triggering growth arrest and cell death. Indeed, pre-clinical testing of small molecule inhibitors of eIF4A such as rocaglates and hippuristanol have shown efficacy in a number of cancer models [11,12,13,14]. The unique enzymatic inhibitory mechanism of BLF1 may therefore offer advantages over conventional toxins for targeted toxin therapy.

We have previously shown that delivery of recombinantly expressed BLF1 into mouse neuroblastoma cells using lipofectamine 3000 (LF3000) leads to cell growth arrest with high potency [15]. LF3000 was used as it increases the potency of toxins in cell lines by around 1000-fold, allowing assessment of activity at low nanomolar concentrations similar to that seen with targeted immunotoxins. In this study we look in greater detail at the anti-proliferative effect of BLF1 in neuroblastoma cells with an emphasis on MYCN amplification status. Amplification of MYCN, a gene belonging to the Myc family of transcription factors, is found in around 50% of advanced stage neuroblastoma patients and is a significant marker of poor prognosis [16,17]. Overexpression of this gene has been shown to primarily increase expression of a number of genes involved in protein synthesis and ribosome biogenesis, making translation initiation a promising target for intervention [18]. We demonstrate that LF3000-mediated delivery of BLF1into cells selectively induces apoptosis in MYCN-amplified neuroblastoma cell lines and preferentially down-regulates the translation of eIF4A dependent proteins (as has been seen with small molecule inhibitors of eIF4A). This highlights the potential for incorporation of BLF1 into targeted toxin design. Additionally, we show that the small molecule inhibitor of eIF4A rocaglamide A (RocA) demonstrates selectivity towards MYCN over-expressing cells, making eIF4A a novel target for neuroblastoma treatment.

## 2. Results

### 2.1. BLF1 Induces Apoptosis in MYCN-Amplified Neuroblastoma

**Figure 1:**
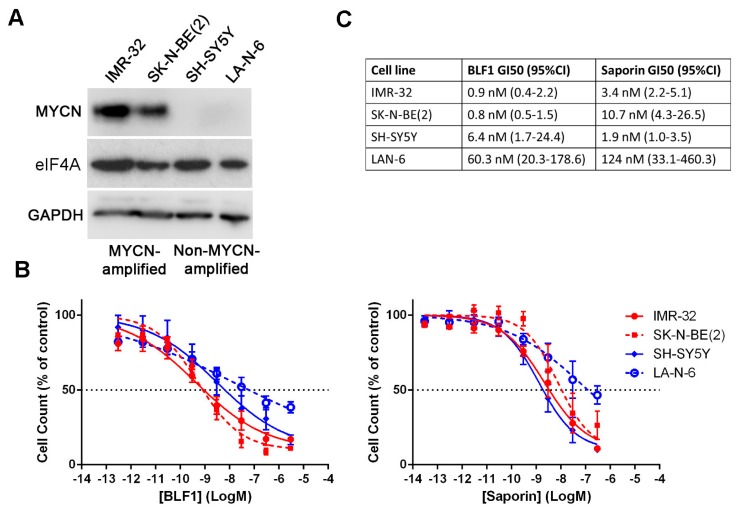
BLF1 causes growth inhibition preferentially in MYCN-amplified cell lines. (**A**) Immunoblot showing protein levels of MYCN and GAPDH in the MYCN-amplified cell lines IMR-32 and SK-N-BE(2), and the non-MYCN-amplified cell lines SH-SY5Y and LA-N-6; (**B**) Alamarblue assay following 72 h titration with BLF1 or saporin in the presence of LF3000. MYCN-amplified cell lines are shown in red and non-MYCN-amplified cell lines are shown in blue (*n* ≥ 3, ± SEM); (**C**) Table showing calculated GI50s and 95% confidence intervals.

To assess the importance of eIF4A in MYCN-driven neuroblastomas, we investigated the effects of eIF4A inactivation by BLF1 on cell growth in cell lines with or without MYCN amplification. BLF1 activity was compared to the ribosome-inactivating protein saporin. Saporin is an enzyme produced in *Saponaria officinalis* seeds that depurinates 28S ribosomal RNA leading to inactivation of the ribosome and a complete block of protein synthesis [19]. This makes saporin highly toxic to all cell types and a good positive control for intracellular protein delivery. The effect of these enzymes on cell growth was tested in four different neuroblastoma cell lines of which two were MYCN-amplified (IMR-32 and SK-N-BE(2)), and two were non-MYCN-amplified (SH-SY5Y and LA-N-6). MYCN expression in the different cell lines was confirmed by immunoblotting which showed substantially higher MYCN levels in the MYCN-amplified cell lines (Figure 1A). Blotting for eIF4A showed that eIF4A levels do not appear to be affected by MYCN expression (Figure 1A). Analysis of growth inhibition following delivery with LF3000 and a 72-h incubation showed that both BLF1 and saporin exhibit similar levels of potency with GI50s in the low nanomolar range (Figure 1B,C). 

**Figure 2:**
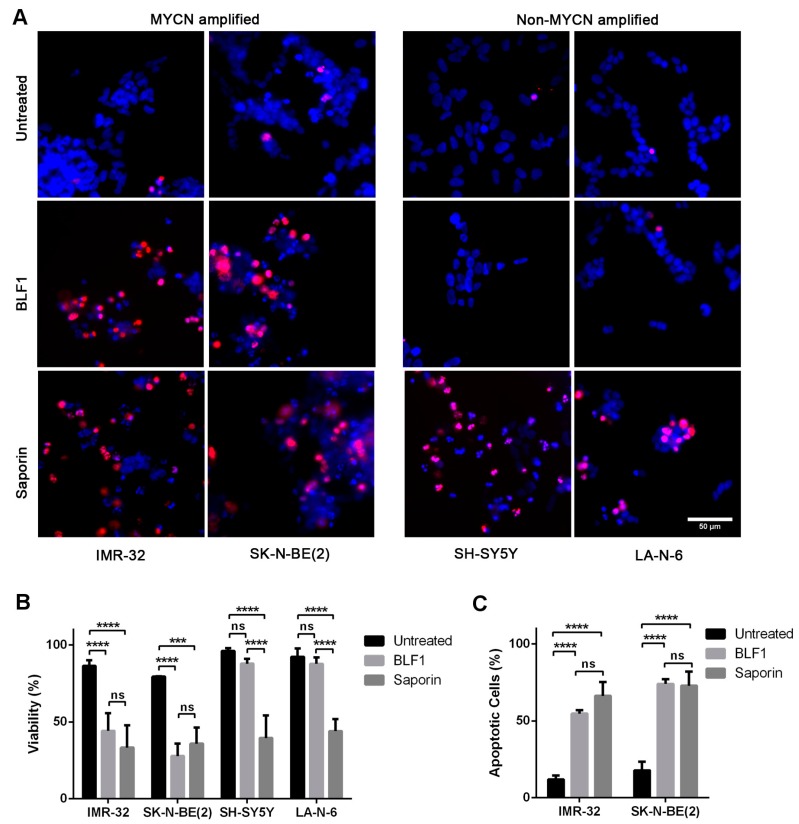
BLF1 induces apoptosis in MYCN-amplified neuroblastoma cell lines. (**A**) Fluorescence microscopy using Hoechst (blue) and propidium iodide (red) staining shows viability of neuroblastoma cell lines following 72 h incubation with 300 nM BLF1 or 300 nM saporin in the presence of LF3000; (**B**) Quantification of A shows that BLF1 exhibits similar cytotoxicity to saporin in MYCN-amplified cell lines but is not significantly cytotoxic to non-MYCN-amplified cells when compared to the untreated control (LF3000 alone), whereas saporin still exhibits high levels of cytotoxicity (*n* = 3, ± SEM). Data was analysed by two-way ANOVA and Tukey’s multiple comparisons test with *** *p* < 0.001 and **** *p* < 0.0001; (**C**) CellEvent Caspase 3/7 activation assay shows that 300 nM BLF1 induces similar levels of apoptosis to saporin (300 nM) following a 72-h incubation in the presence of LF3000 (*n* = 3, ± SEM). Data was analysed by two-way ANOVA and Tukey’s multiple comparisons test with **** *p* < 0.0001; ns: non significant.

The effect of BLF1 on neuroblastoma cells was investigated in more detail by analysing cell viability using the nuclear dyes propidium iodide (which stains dead cells) and Hoechst 33342 (which stains all cells). The toxins were added to cells at a high concentration of 300 nM as this causes maximal growth inhibition of all the cell lines (Figure 1B). Staining following incubation for 72 h with 300 nM BLF1 or 300 nM saporin in the presence of LF3000 showed that BLF1 caused cell death at a similar level to saporin in MYCN-amplified cells (Figure 2A,B). However, in MYCN-non-amplified cells, BLF1 had no significant effect on cell viability, as evidenced by the lack of propidium iodide staining and normal nuclear morphology, whereas saporin still caused cell death (Figure 2A,B). This suggests that BLF1 is preferentially cytotoxic to MYCN amplified cell lines, but is only cytostatic in non-MYCN-amplified cells. Analysis of the mechanism of cell killing in the IMR-32 and SK-N-BE(2) MYCN-amplified cells revealed that both BLF1 and saporin cause caspase 3/7 activation and induce apoptosis (Figure 2C). Of note, SK-N-BE(2) cells have a non-functional p53 mutation which suggests that the induction of apoptosis occurs independently of p53 [20]. This is of potential therapeutic benefit because inactivating p53 mutations are often found in refractory tumours following relapse [21]. 

### 2.2. The Cytotoxic Effect of eIF4A Inhibition in Neuroblastoma Is Directly Dependent on MYCN Expression

**Figure 3:**
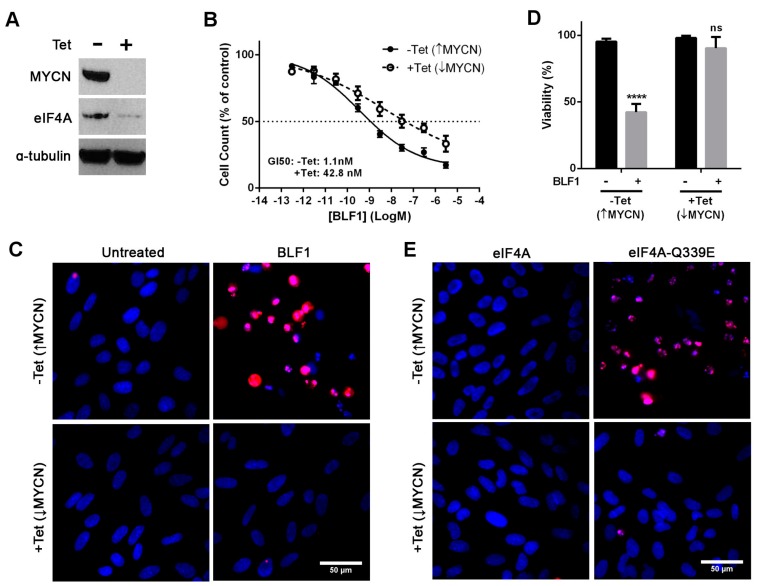
BLF1 exhibits increased potency in SHEP-21N cells expressing MYCN. (**A**) Immunoblot showing protein levels of MYCN, eIF4A and a loading control α-tubulin in SHEP-21N cells following 72 h of incubation in the presence or absence of tetracycline; (**B**) AlamarBlue assay following 72-h titrations with BLF1 in the presence of LF3000 demonstrates increased growth inhibition in SHEP-21N cells expressing MYCN (*n* = 3, ± SEM); (**C**) Fluorescence microscopy using Hoechst (blue) and propidium iodide (red) staining shows viability of SHEP-21N cells following 72-h incubation with 300 nM BLF1 in the presence of LF3000; (**D**) Quantification of the panel C results shows that BLF1 is significantly cytotoxic to SHEP-21N cells expressing MYCN but has no significant effect on cells not expressing MYCN when compared to the untreated control (LF3000 alone) (*n* = 3, ± SEM). Data was analysed by multiple unpaired t-tests with **** *p* < 0.0001; ns: non significant. (**E**) Fluorescence microscopy using Hoechst and propidium iodide staining shows viability of SHEP-21N cells following 72 h incubation with 1 μM eIF4A or eIF4A-Q339E dominant-negative mutant in the presence of LF3000.

To confirm that cytotoxicity caused by BLF1 is MYCN dependent, the SHEP-21N cell line was used. This neuroblastoma cell line constitutively expresses high levels of MYCN which can be repressed by the addition of tetracycline [22]. Down-regulation of MYCN after a 72-h incubation with tetracycline was confirmed via immunoblotting (Figure 3A). Interestingly, down-regulation of MYCN also caused a decrease in levels of eIF4A, suggesting a regulatory role of MYCN in eIF4A expression (Figure 3A). This is in agreement with a previous study which utilised the SHEP-21N cell line and showed that MYCN up-regulates eIF4A as well as a number of other proteins involved in ribosome biogenesis and protein synthesis [18].

Analysis of growth inhibition showed that BLF1 efficacy is decreased in the SHEP-21N cell line following tetracycline-induced down-regulation of MYCN (GI50: 1 nM without tetracycline and 42 nM with tetracycline) (Figure 3B). Propidium iodide staining showed that 300 nM BLF1 was only cytotoxic to the cells expressing high levels of MYCN (Figure 3C,D). BLF1 causes cytotoxicity by deamidation of a specific glutamine residue (Gln-339) in the eIF4A translation factor to glutamic acid [5]. This creates a dominant negative eIF4A mutant (eIF4A-Q339E) that can bind to mRNA and stall ribosome initiation complexes. To confirm that the cytotoxicity observed is due to eIF4A inhibition, the Gln-339 eIF4A mutant was generated and delivered into SHEP-21N cells using LF3000. Again, cytotoxicity was only observed in cells expressing high levels of MYCN (Figure 3E). No effect on cell viability was seen after the addition of the native eIF4A protein (Figure 3E). 

**Figure 4:**
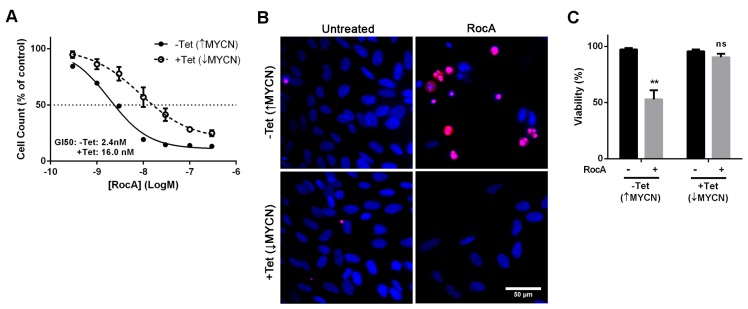
RocA exhibits increased potency in SHEP-21N cells expressing MYCN. (**A**) AlamarBlue cell assay shows more potent growth inhibition in SHEP-21N cells expressing MYCN, following 72 h titrations with RocA (*n* = 3, ± SEM); (**B**) Fluorescence micrographs showing cell viability following 72 h of incubation with 100 nM RocA. All nuclei are stained with Hoechst (blue) whereas the nuclei of dead cells are stained with propidium iodide (red); (**C**) Quantification of the panel C results shows that RocA is significantly cytotoxic to SHEP-21N cells expressing MYCN but has no effect on viability of cells not expressing MYCN when compared to the untreated control (DMSO alone) (*n* = 3, ± SEM). Data was analysed by multiple unpaired t-tests with ** *p* < 0.01; ns: non significant.

The effect of small molecule inhibition of eIF4A in the SHEP-21N cell line was then tested to independently confirm the suitability of eIF4A as a therapeutic target. Rocaglamide A (RocA) is a small molecule isolated from Aglaia plants that has recently been shown to convert eIF4A into a sequence selective translational repressor [23]. Assessment of growth inhibition following titrations of RocA revealed that MYCN expressing SHEP-21N cells were over six times more sensitive compared to the tetracycline-treated SHEP-21N with MYCN being suppressed (GI50: 2.4 nM without tetracycline and 16 nM with tetracycline) (Figure 4A). Co-staining with propidium iodide and Hoechst revealed that 100 nM RocA was cytotoxic to cells expressing MYCN but had no significant effect on viability of cells with suppressed MYCN (Figure 4B,C). Taken together, these data indicate that the cytotoxicity observed in cells expressing high levels of MYCN is due to inhibition of eIF4A.

### 2.3. BLF1 Down-Regulates MYCN and Other Oncogenic Proteins

**Figure 5:**
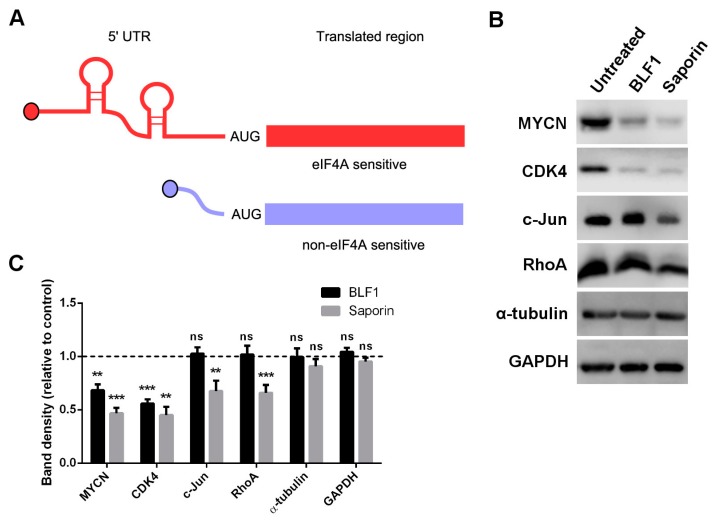
BLF1 down-regulates protein levels of MYCN and other eIF4A sensitive transcripts. (**A**) Schematic showing eIF4A sensitive (red) and non-eIF4A sensitive (blue) mRNAs. eIF4A sensitive mRNAs contain long and highly structured 5′ UTRs that require the RNA helicase of eIF4A to unwind secondary structures such as G-quadruplexes for scanning by the 43S pre-initiation translation complex. These coding transcripts usually encode for oncogenes and pro-survival proteins [10]; (**B**) Immunoblot showing protein levels of MYCN, CDK4, c-Jun, RhoA, α-tubulin and GAPDH in IMR-32 cells following a 24-h incubation with 30 nM BLF1 or saporin in the presence of LF3000; (**C**) Quantification of the panel B results shows the high level of down-regulation of MYCN and CDK4 following treatment with either toxin, whereas only saporin down-regulates the eIF4A insensitive transcripts c-Jun and RhoA. No effect on housekeeping genes GAPDH or α-tubulin was seen. Protein levels are shown as a ratio of the untreated control (LF3000 alone) (*n* ≥ 3, ± SEM). Data was analysed by multiple one-sample t-tests to a normalised control of 1 with ** *p* < 0.01 and *** *p* < 0.001; ns: non significant.

Pharmacological inactivation of eIF4A has been shown to down-regulate a number of oncogenic proteins which are encoded by mRNAs containing long and highly structured 5′ UTRs (Figure 5A) [10]. We therefore examined the effect of eIF4A inhibition on protein levels of MYCN and the cell-cycle regulator CDK4, which has previously been shown to be sensitive to the small molecule eIF4A inhibitors silvestrol and hippuristanol [13,24]. Two proteins that, according to ribosome-footprinting, should be insensitive to eIF4A inhibition, c-Jun and RhoA, were also used [10]. GAPDH and α-tubulin were used as controls, as their long half-lives mean levels should not be significantly affected by protein synthesis inhibition over 24 h. IMR-32 cells were incubated with 30 nM of protein as this concentration showed similarly high toxic activity for both BLF1 and saporin in growth assays (Figure 5B). Immunoblotting following a 24 h incubation with BLF1 or saporin in the presence of LF3000 revealed a dramatic decrease in MYCN and CDK4 protein levels after treatment with either enzyme (Figure 5B,C). However, only saporin caused down-regulation of the eIF4A-insensitive proteins c-Jun and RhoA. No effect was seen on levels of the house-keeping proteins GAPDH and α-tubulin. This shows that, as with small molecule inhibitors of eIF4A, BLF1 is able to preferentially down-regulate a subset of proteins. The specific down-regulation of MYCN provides a novel mechanistic insight into the cytotoxic activity of BLF1.

### 2.4. BLF1 Has No Effect on Primary Mouse Fibroblasts

**Figure 6:**
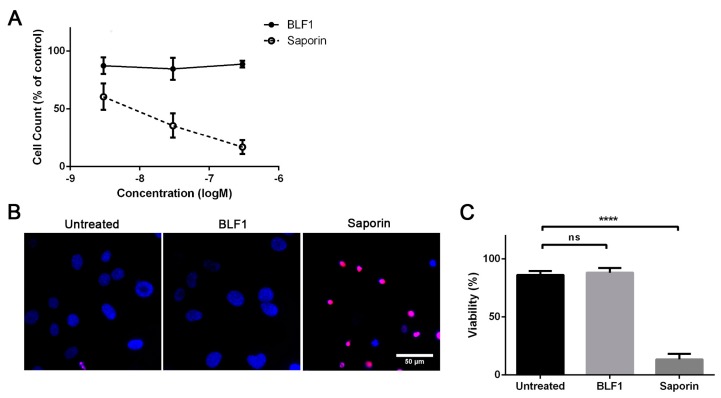
BLF1 has no effect on viability of primary mouse fibroblasts. (**A**) AlamarBlue assay following titrations of BLF1 and saporin in the presence of LF3000 in quiescent mouse fibroblasts shows that BLF1 has no effect on cell numbers whereas saporin causes a large reduction (*n* = 3, ± SEM); (**B**) Fluorescence microscopy images showing Hoechst 33342 (blue) and propidium iodide (red) staining following treatment of mouse fibroblasts with 300 nM BLF1 or saporin in the presence of LF3000; (**C**) Quantification of B shows that BLF1 has no effect on cells whereas saporin causes a significant amount of cell death (*n* = 3, ± SEM). Data was analysed by one-way ANOVA and Dunnet’s multiple comparisons test with **** *p* < 0.0001; ns: non significant.

BLF1 shows cytostatic activity in non-MYCN-amplified cells which suggests that it may also have reduced efficacy in non-dividing healthy cells. This is an important characteristic when considering viable therapies for cancer treatment. To test this, primary fibroblasts were cultured from the mouse ear, grown until fully confluent and cultured in low serum media. This has previously been shown to inhibit cell division and cause cells to enter a quiescent state [25]. Analysis of cell numbers showed that BLF1 had no effect on viability, whereas saporin was still able to potently induce cell death (Figure 6A). This was confirmed by Hoechst 33,342 and propidium iodide staining which showed that 300 nM saporin caused nuclear condensation characteristic of apoptosis, whereas 300 nM of BLF1 had no effect on nuclear morphology (Figure 6B,C). The lack of efficacy of BLF1 in non-dividing cells suggests a wide therapeutic window that will be of benefit for future therapy development.

## 3. Discussion

In this paper, we have shown that inactivation of eIF4A by BLF1, following intracellular delivery with LF3000, inhibits the growth of neuroblastoma cells with high potency. Furthermore, this toxin exhibits increased efficacy and a specific cytotoxic action in MYCN amplified cells. Downregulation of MYCN via siRNA has previously been shown to induce caspase 3 activation and apoptosis in MYCN amplified neuroblastoma [26]. Caspase 3 activation and induction of apoptosis in a MYCN dependent manner was also observed following BLF1 treatment, demonstrating selective cytotoxicity of this toxin towards MYCN-amplified neuroblastoma cell lines. The exact mechanism by which BLF1 exerts cytotoxicity towards MYCN-amplified cells but is only cytostatic towards non-MYCN-amplified cells remains unclear. Levels of eIF4A do not appear to differ between MYCN-amplified and non-MYCN-amplified cells, which suggests that cytotoxicity is due to inhibition of cellular processes necessary for cell survival in a MYCN-driven setting and not an increased dependence on eIF4A itself. Ribosome profiling has previously highlighted hundreds of transcripts that are sensitive to eIF4A inhibition [10]. Importantly, a number of eIF4A sensitive transcripts have been shown to be critical for MYCN-dependent cell growth, including the p53 inhibitor MDM2 [27], the pro-survival protein BCL2 [28] and the transcription factor B-MYB [29]. BLF1 caused a down-regulation of the eIF4A-sensitive oncogenic protein CDK4 which has previously been shown to be important for maintaining an undifferentiated phenotype in neuroblastoma cell lines [30]. Inactivation of eIF4A appears to concomitantly target several key pathways involved in proliferation and cell survival of MYCN amplified neuroblastoma, making eIF4A a highly attractive target for the treatment of MYCN-amplified tumours.

The highly potent selective toxicity exhibited by BLF1 upon entry into the cytosol highlights this toxin as a potential future therapeutic. Commonly used toxins in immunotoxin therapies, such as saporin, work by the global inhibition of protein synthesis which leads to cell death in both healthy and transformed cells [31]. Off-target toxicity due to non-specific uptake of toxin by healthy cells has led to dose-limiting side effects in a number of trials and limited the success of this form of treatment [1]. BLF1 induced cell death in MYCN-amplified neuroblastoma with high potency similar to saporin, but had only a cytostatic effect on non-MYCN amplified cells at the concentrations tested. Additionally, no effect was seen following BLF1 treatment of quiescent fibroblasts. This makes BLF1 a promising candidate for immunotoxin development as it displays a degree of selectivity towards rapidly dividing cells. This may help to reduce common side effects observed during immunotoxin treatment and increase the therapeutic window. Although validated in MYCN-driven neuroblastoma cell lines, BLF1 may also be of use in more common c-Myc-driven tumours, as has been seen with small molecule inhibitors of eIF4A. This would greatly increase the applicability of this enzyme for cancer treatment as c-Myc is one of the key factors driving cancerous growth [32]. Further investigation of BLF1 as an immunotoxin and targeting of this enzyme to MYCN-amplified neuroblastoma or other Myc-driven cancers will therefore be of high interest.

MYCN-amplified neuroblastoma is a high-risk disease with very few treatment options and poor outcome. A common feature of these cancers is the acquisition of chemo-resistance and relapse, making identification of novel targets for treatment of this disease of high importance [17]. The selective cytotoxic effects of BLF1 and RocA observed in MYCN-expressing SHEP-21N cells suggests that future immunotoxins and eIF4A small molecule inhibitors have potential as future therapeutics in the treatment of neuroblastomas exhibiting MYCN amplification.

## 4. Materials and Methods 

### 4.1. Recombinant Proteins, Toxins and Small Molecules

Saporin from *Saponaria officinalis* seeds and Rocaglamide A were obtained commercially from Sigma. Recombinant hexahistidine-tagged fusions of BLF1 and eIF4A encompassing amino-acids 20-406 were purified using Ion Metal Affinity Chromatography on TALON-Cobalt (Clontech, Fremont, CA, USA) and S200 gel filtration (GE Healthcare, Little Chalfont, UK) as described previously [5].

### 4.2. Cell Culture

SHEP-21N cells were a gift from Prof. Manfred Schwab, and IMR-32 cells were a gift from Prof. Peter Andrews. SK-N-BE(2) and LA-N-6 cells were obtained from the Childrens Oncology Group Cell Culture Repository, and SH-SY5Y cells were obtained from Sigma. SHEP-21N, IMR-32, SK-N-BE(2) and LA-N-6 cells were grown in RPMI media (Life Technologies, Carlsbad, CA, USA) supplemented with 10 per cent Fetal Bovine Serum (FBS) (Life Technologies). SH-SY5Y cells were grown in a 1:1 mix of MEM (Life Technologies) and F12 nutrient mix (Life Tehcnologies) supplemented with 15 per cent FBS and 1 per cent non-essential amino acids (Life Technologies). Cells were maintained at 37 °C, 5 per cent CO_2_. 

For primary mouse fibroblast cultures, C57BL/6 mice were sacrificed using a humane method as listed in Schedule 1 of the Animal (Scientific procedure) Act 1986. Small segments of tissue (approximately 3 mm^2^) were taken from the ear and placed in Hank’s balanced salt solution (HBSS) (Life Technologies). The tissue was then diced into small pieces and incubated with 2000 U/mL collagenase XI (Sigma) for 25 min at 37 °C. Following washing in HBSS, 0.05 per cent trypsin was added and the tissue was incubated for 20 min at 37 °C. The supernatant was discarded, and the tissue was re-suspended in fibroblast media (DMEM + 1 per cent NEAA + 1 per cent penicillin/streptomycin (P/S) (Sigma) + 10 per cent FBS), followed by trituration to break up cell aggregates. The suspension was plated in 3 cm dishes and incubated at 37 °C for 3 to 4 days to allow cell growth. Cells were expanded to a 25 cm^2^ flask followed by a 75 cm^2^ flask. Sub-culturing was carried out every 3–4 days when spent media was replaced or cells were passaged. For viability studies, cells were seeded in a 96-well plate and grown for 3–4 days until fully confluent. The media was then changed to low serum (0.1 per cent FBS) and cells were cultured for a further 1–2 days before treatment with proteins.

### 4.3. Cell Treatment and Protein Transduction

For pharmacological treatment, cells were seeded in 96-well plates at a density of 1 × 10^4^ cells or in 48-well plates at a density of 2.5 × 10^4^ cells and left for at least 5 h to attach. For immunoblotting experiments, cells were left for 24 h before treatment. Protein was delivered into cells by lipofection as described previously [15]. Briefly, protein was diluted to 10 times the final concentration in Optimem (Life Technologies) before the addition of LF3000 (Life Technologies) in a 1/50 dilution. The mix was incubated at 20 °C for 20 min before adding to cells in a 1/10 dilution to give the final concentrations.

### 4.4. Growth Inhibition Assay

Growth inhibition was measured using the alamarBlue assay (Thermofisher, Waltham, MA, USA) according to manufacturer’s instructions. Following incubation with drugs or protein, the alamarBlue reagent was added directly to cells in a 1/10 dilution. Cells were incubated at 37 °C for 4 h before fluorescence was measured at 560 nm excitation/590 nm emission using a Fluorskan Ascent plate reader (Thermo Fisher Scientific, Loughborough, UK). GI50s were calculated as the concentration necessary to cause 50 per cent growth inhibition compared to vehicle (DMSO for RocA or LF3000 for protein) treated cells after normalisation to a blank control (cell-free medium with alamarBlue reagent).

### 4.5. Fluorescence Microscopy

For microscopy assays, cells were seeded in μClear 96-well plates (Greiner, Kremsmünster, Austria) for greater resolution and incubated for 72 h following treatment before imaging. Viability of cells was assessed by the addition of 1 μg/mL Hoechst 33,342 (Life Technologies), which stains the DNA of all cells, and 0.5 μg/mL Propidium iodide (Life Technologies), which only stains the DNA of dead cells. Cells were then incubated at 37 °C for 15 min before imaging using an epifluorescence microscope (CMIRB, Leica, London, UK) at a 40× objective. Experiments were performed in duplicate with at least 4 images taken per well. Cells were counted using ImageJ and the percentage of Propidium iodide to Hoechst stained cells was calculated to give viability.

Apoptosis was assessed using the CellEvent Caspase 3/7 Green detection kit (Life Technologies) according to manufacturer’s instructions. The CellEvent reagent was added to cells at a final concentration of 5 μM and cells were incubated at 37 °C for 15 min. Hoechst 33,342 (1 μg/mL) was then added and cells were incubated for a further 15 min before imaging. Images were taken at a 40× objective and the number of apoptotic cells was then calculated as a percentage of the total cell number. Experiments were performed in duplicate and at least 4 images were taken per well.

### 4.6. Immunoblotting

Following 24 h treatment, cells were washed once with cold PBS before lysis using RIPA buffer (50 mM Tris-HCl pH 7.5, 150 mM NaCl, 1 mM EDTA, 1 mM EGTA, 0.1 per cent SDS, 0.5 per cent sodium deoxycholate and 1 per cent NP-40). Protein concentration was measured using the DC assay (Biorad, Hercules, CA, USA) according to manufacturer’s instructions. Lysates were run on 12 per cent Bis-Tris sodium dodecyl sulphate-polyacrylamide gel electrophoresis (SDS-PAGE) gels (Life Technologies) and protein was transferred to a polyvinylidene difluoride membrane (Biorad) before probing with antibodies. Primary antibodies used include MYCN (Santa-Cruz: sc-56729, 1:500 [33]), eIF4A1 (Cell Signalling: 2490, 1:1000 [23]), CDK4 (Abcam: ab108357, 1:1000 [34]), c-Jun (Santa-Cruz: sc-74543, 1:500 [35]), RhoA (Santa-Cruz: sc-418, 1:500 [36]), GAPDH (Life Technologies: AM4300, 1:5000 [37]) and α-tubulin (Sigma: T6074, 1:5000 [38]). Following incubation with peroxidase conjugated sheep anti-mouse or donkey anti-rabbit secondary antibodies (1:24,000, GE Healthcare), proteins were visualised using the SuperSignal West Dura ECL reagent (Thermo Scientific, Waltham, MA, USA) by X-ray film exposure with signals quantified using ImageJ after film scanning. 

### 4.7. Statistical Analysis

Data were expressed as mean ± SEM and specific statistical tests are indicated in the figure legends. Statistical analysis was carried out using Graphpad Prism 6. A *p*-value <0.05 was considered statistically significant. For single comparisons, unpaired t-tests were used. For single comparisons to a normalised control the one-sample t-test was used. For multiple comparisons, data was analysed by either one-way ANOVA followed by the Dunnet’s method for comparison to a single control sample, or by two-way ANOVA followed by the Tukey range test.
